# Mechanistic Studies of Arene–Ruthenium(II) Complexes with Carbothioamidopyrazoles as Alternative Cancer Drugs

**DOI:** 10.3390/molecules28093969

**Published:** 2023-05-08

**Authors:** Paweł Hikisz, Ewelina Namiecińska, Piotr Paneth, Elzbieta Budzisz

**Affiliations:** 1 Department of Oncobiology and Epigenetics, Faculty of Biology and Environmental Protection, University of Lodz, Pomorska 141/143, 90-236 Lodz, Poland; pawel.hikisz@biol.uni.lodz.pl; 2 Department of the Chemistry of Cosmetic Raw Materials, Medical University of Lodz, Muszynskiego 1, 90-151 Lodz, Poland; ewelina.namiecinska@umed.lodz.pl; 3 Institute of Applied Radiation Chemistry, Department of Chemistry, Technical University of Lodz, Zeromskiego 116, 90-924 Lodz, Poland; piotr.paneth@p.lodz.pl

**Keywords:** anticancer activity, molecular docking, arene–ruthenium(II) complexes, carbothioamidopyrazoles

## Abstract

Arene–ruthenium(II) complexes with carbothioamidopyrazoles at the C-2 and C-5 positions have been recognized as chemotherapeutic agent alternatives to cisplatin and its oxaliplatin analogs. The aim of this study was to continue research on the biological aspect of arene–ruthenium(II) complexes and their anticancer activity. The present paper includes an additional 12 new tumor cells, analyzed by MTT, and employs a series of extended bioassays to better understand their potential mechanism of antitumor activity. The following tests were conducted: membrane permeability studies, intramolecular reactive oxygen and nitrogen species (ROS/RNS) assays, mitochondrial potential changes, DNA analysis by comet assay using the electrophoresis method, measurement of cleaved PARP protein levels, and determination of apoptotic and necrotic cell fractions by fluorescence microscopy. Additionally, the article presents lipophilicity studies based on RP-TLC and molecular docking studies. We hope that the presented data will prove useful in practical treatment, especially for patients with cancer.

## 1. Introduction

Anticancer platinum drugs have been associated with strong toxicity to healthy cells and acquired or inherited cancer cell resistance, prompting the search for alternative medical solutions. Complexes with metal ions other than platinum often employ different mechanisms and targets of action to achieve their anticancer effects; they have a different pharmacological profile and often demonstrate less toxic effects on healthy cells [1]. There is little information on the molecular basis or mechanisms of action of these compounds. Metal complexes employ a range of anticancer mechanisms, and their overall activity results from several extracellular and intracellular interactions. Currently, biological evaluations of the anticancer activity of metal compounds tend to be based around interference analysis of protein–protein interactions, enzymatic inhibition (for example topoisomerases or multiprotein proteasomes), and redox modulation as well as chromatin and histone targeting. Mitochondria are also becoming important targets in modern chemotherapy [2,3,4].

The tested copper complexes, including those containing pyrazole derivatives of thiosemicarbazone, imidazole, and pyridine, have shown good selectivity against tumors, and some have negated cisplatin resistance [5,6,7,8,9]. Ruthenium complexes including ruthenium(III) ions are also the subject of research and they might potentially be used in drug formulation. Compounds such as NAMI-A, KP1019, and NKP-1339 have undergone phase I and II clinical trials for combating metastatic solid tumors, refractory tumors, lung cancer, colorectal cancer, and neuroendocrine tumors [10,11,12].

**Scheme 1:**
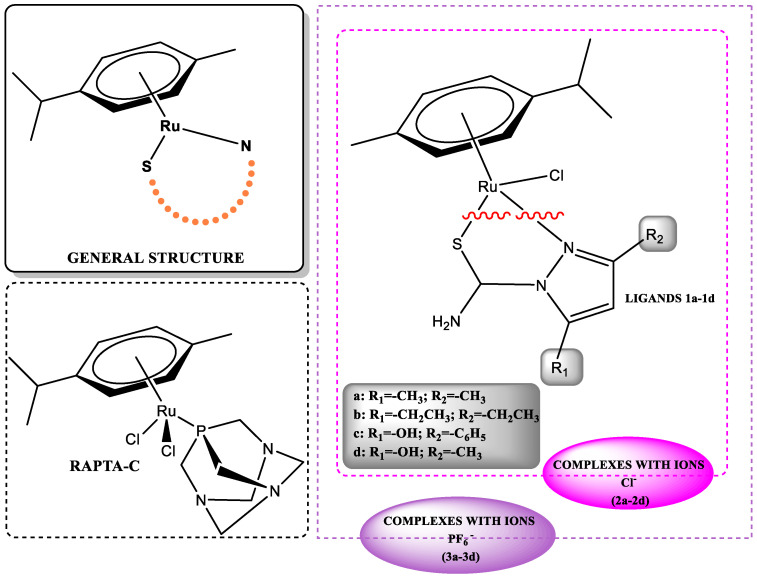
Characteristics of general structure of RAPTA–C and arene–ruthenium(II) complexes with carbothioamidopyrazoles **2a**/**3a**–**2d**/**3d** and its ligands **1a**–**1d**.

In addition to ruthenium(III) complexes, the interesting ruthenium(II) complexes have also appeared. These possess an areno substituent which effectively stabilizes the structure and oxidation state of the compound, increasing its hydrophobicity and thus facilitating absorption through biological membranes [13,14]. RAPTA compounds with the general formula [Ru(*η*^6^-areno)Cl_2_(PTA)](PTA = 1,3,5-triazo-7-phosphaadamantane) are interesting representatives. They are characterized by low in vitro cytotoxicity, especially RAPTA-C (Scheme 1), which showed a significant reduction in primary tumors in preclinical models of ovarian and colorectal cancer. Like platinum(II) compounds, complexes with ruthenium ions occur at different oxidation levels (from +II to +IV) under physiological conditions and imitate iron(III) ions by binding molecules such as albumin or transferrin; they also demonstrate comparable ligand exchange rates. In addition, ruthenium ions possess an octahedral coordination sphere that differs from the flat geometry of platinum(II), implying a different mechanism of action. Thus, the compounds might be potentially applied in anticancer therapy [15].

Our team has already documented antitumor activity for HL-60, NALM-6, and WM-115 cancer cells of *p*-cymenoruthenium(II) complexes with pyrazole ligands **2a**/**3a**–**2d**/**3d** (Scheme 1), and satisfactory results have been reported for the melanoma line. The complexes turned out to be twice as potent as the previously-used cisplatin [16]. The compounds have previously been studied with regard to their antimicrobial action; they appeared to demonstrate an adjunctive effect to some antibiotics, which could be an interesting addition to adjuvant therapy, especially for patients weakened after cancer therapy [17]. 

The present paper extends this line of research to include another twelve new tumor cells analyzed by the MTT method. It also provides an insight into the potential mechanism of the antitumor activity of the compounds based on extended bioassays. The following tests were conducted: membrane permeability studies, intramolecular reactive oxygen and nitrogen species (ROS/RNS) assays, mitochondrial potential changes, DNA analysis by comet assay using the electrophoresis method, measurement of cleaved PARP protein levels, and determination of apoptotic and necrotic cell fractions by fluorescence microscopy. In addition, lipophilicity studies were performed by RP-TLC and molecular docking.

## 2. Results

### 2.1. Cytotoxicity

Cytotoxicity was assessed in vitro against fifteen human primary cancer cell lines using the MTT test, one of the most commonly used tests for evaluating cytotoxic activity against metabolically active cells. The cytotoxic effect was assessed after 48 h incubation with a wide range of concentrations of investigated compounds. This experimental approach allows cells to undergo mitosis in the presence of the investigated compounds, thus providing an insight into their inhibitory effect on cell proliferation. Determined IC_50_ concentrations after 24 h incubation are shown in Table 1. As expected from our previous studies [16], ligands **1a**–**1d** showed almost no cytotoxic properties. The vast majority of these compounds were completely inactive against cancer cells in the wide range of used concentrations: a slight decrease in cell survival was observed in the case of Ishikawa, Hec-1A, WM-115, and NALM-6 cells. 

Ruthenium complexes **2a**–**3d** were characterized by various biological effects in relation to the cancer cells used. A very low cytotoxic effect was observed for A549, HCC39, and Caco2. Generally, the remaining cell lines demonstrated dose-dependent inhibition of cell growth and a significant decrease in cell viability with increasing concentrations of ruthenium complexes. Noticeably, compounds **2c** and **3c** seem to be particularly attractive in terms of potential anticancer properties, with IC_50_ < 50 µm/l for seven and six cancer cell lines, respectively. Although the remaining ruthenium complexes demonstrated a slightly smaller spectrum of activity against cancer cells, their anticancer activity against some lines was very satisfactory. Interesting results were undoubtedly obtained for the WM-115 line, which was very sensitive to compounds **2d** and **3c**. It should be emphasized that for the **2d** derivative, the IC_50_ was lower than that of the cisplatin reference compound.

In the first stage, the analysis of the anticancer activity of ruthenium derivatives examined their cytotoxic properties against fifteen cancer cell lines. This study was aimed at, inter alia, screening the cytotoxic properties of the tested compounds and selecting the most active ones for further research. The cutoff was selected as IC_50_ = 55 µM. The compounds that demonstrated low biological effectiveness (IC_50_ above 55 µM) and clearly did not inhibit the cell profiling of a given cancer line were not analyzed for DNA damage, apoptosis, or PARP degradation. Thus, ten cell lines were selected for further research: NALM6, HEC1A, WM-15, COLO205, Hep 3b, HT29, HCT116, SW620, Ishikawa, and MCF7, as well as compounds **2a**, **2b**, **2c**, **2d**, **3a**, **3b**, and **3c** which were used for individual cell lines. The selected cell lines and compounds were most suitable for studying antitumor activity and thus for determining their potential value as chemotherapeutic agents. In addition to the MTT study, the ten cancer cell lines were subjected to cytotoxicity and compared to two reference compounds—cisplatin and NAMI -A. The results are presented in Table 2.

Importantly, for the selected and most active ruthenium derivatives **2a, 2b, 2c, 2d, 3a, 3b**, and **3c**, a cytotoxicity test was performed on a normal, immortalized human microvascular endothelial cell line HMEC1. The results are presented in Table 3. Regarding the potential application of the tested ruthenium derivatives as chemotherapeutic agents, it is important to note that the analyzed compounds were characterized by very low cytotoxicity, clearly weaker than that observed in neoplastic cells.

### 2.2. Reactive Oxygen/Nitrogen Species

Reactive oxygen/nitrogen species (ROS/RNS) play a key role in basic biological processes occurring in the human body, including in the proper functioning of many cellular processes. ROS production is an inseparable part of the oxygen metabolism of cells. The imbalance between the production of ROS/RNS and the efficiency of antioxidant systems leads to oxidative stress, which results in damage to important cellular macromolecules, i.e., DNA, proteins, and lipids. It is assumed that ROS generation is one of the key mechanisms of anticancer activity of commercially available chemotherapeutic agents. ROS selectively generated in cancer cells damage the DNA and organelles of these cells, ultimately leading to their apoptosis [18]. 

**Figure 1:**
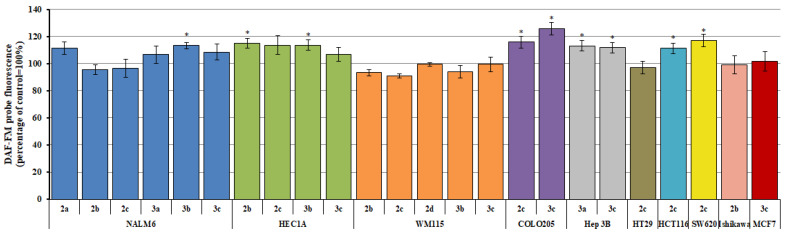
Relative amounts of RNS (DAF-FM probe) generated in NALM6, HEC1A, WM115, COLO 205, Hep 3B, HT29, HCT116, SW620, Ishikawa, and MCF7 treated with investigated compounds. * Results represent mean ± SD of data from three individual experiments, each performed with at least eight repeats; * *p* < 0.05 versus control.

In order to assess the pro-oxidative properties of the analyzed derivatives, three fluorescent probes were used: DAF-FM, DHET, and H_2_DCFDA, which preferentially monitor the increase in the generation of nitric oxide, superoxide O_2_^•−^, and hydrogen peroxide H_2_O_2_ in cells, respectively. The cells were exposed for 24 h to the IC_50_ concentration of complexes and then the level of RNS/ROS was measured with the appropriate fluorescent probe. Definitely the smallest changes in the level of RNS generation were found for the DAF-FM probe (nitrogen peroxide) (Figure 1). The results may suggest that RNS play hardly any role in shaping the cytotoxic properties of the analyzed derivatives. The greatest changes were observed after treatment with compound **3c** for COLO205—about a 25% increase in nitrogen peroxide vs. control. In the case of the remaining cells, the changes were up to 15%.

**Figure 2:**
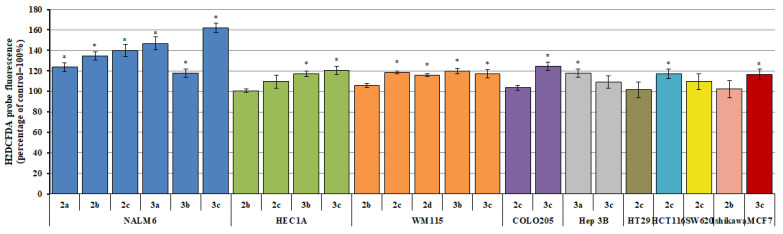
Relative amounts of ROS (H_2_DCFDA probe) generated in NALM6, HEC1A, WM115, COLO 205, Hep 3B, HT29, HCT116, SW620, Ishikawa and MCF7 treated with investigated compounds. * Results represent mean ±SD of data from three individual experiments, each performed with at least eight repeats; * *p* < 0.05 versus control.

The greatest changes were observed for the H_2_DCFDA probe, regarding the generation of hydrogen peroxide in cancer cells. The results are presented in Figure 2. Treatment with the test compounds resulted in a significant increase in hydrogen peroxide content in seven out of ten cancer lines used in the experiment. The NALM6 line remained the most sensitive, with a 20–60% increase in ROS compared to controls, depending on the used derivative. In the case of the remaining cells, the increase in ROS was approximately 20–25%.

**Figure 3:**
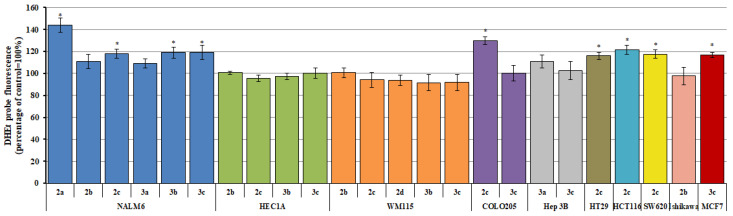
Relative amounts of ROS (DHEt probe) generated in NALM6, HEC1A, WM115, COLO 205, Hep 3B, HT29, HCT116, SW620, Ishikawa, and MCF7 treated with investigated compounds. * Results represent mean ±SD of data from three individual experiments, each performed with at least eight repeats; * *p* < 0.05 versus control.

Slightly smaller changes were observed for the DHET probe (Figure 3) and the detection of superoxide generation. Again, NALM6 were the most sensitive cells, where the generated ROS reached the highest level. However, the observed changes were already about 20% smaller than those noted for hydrogen peroxide. In the case of the remaining cancer lines, where statistically significant changes in the level of ROS were observed, hydrogen peroxide increased by about 20–35%.

### 2.3. Plasma Membrane Fluidity 

The oxidative stress generated by the analyzed derivatives may also induce disturbances in the proper functioning of the cellular plasma membrane. As a result of free radical reactions, polyunsaturated fatty acids are oxidized, which in turn leads to the formation of structurally modified and damaged lipid molecules. Changes in lipid peroxidation were observed using two fluorescent probes, DAUDA and TMA-DPH, located at different depths of the lipid bilayer. TMA-DPH is incorporated in the surface area of the outer monolayer, while DAUDA penetrates into the deeper hydrophobic area of the membrane. 

**Figure 4:**
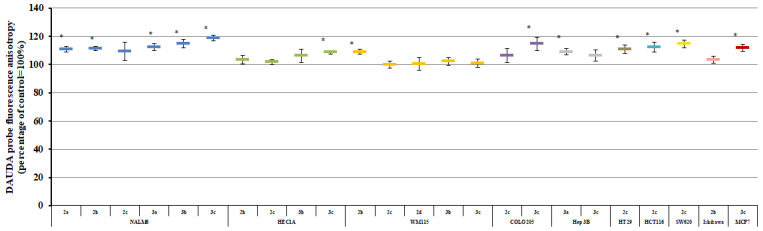
Fluorescence anisotropy of DAUDA label in NALM6, HEC1A, WM115, COLO205, Hep 3B, HT29, HCT116, SW620, Ishikawa, and MCF7 treated with investigated compounds. * Results represent mean ±SD of data from three individual experiments, each performed with at least six repeats; * *p* < 0.05 versus control.

**Figure 5:**
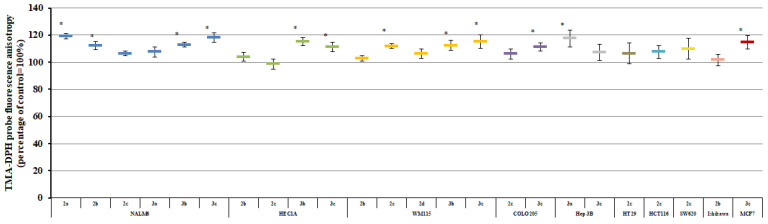
Fluorescence anisotropy of TMA-DPH label in NALM6, HEC1A, WM115, COLO205, Hep 3B, HT29, HCT116, SW620, Ishikawa, and MCF7 treated with investigated compounds. * Results represent mean ± SD of data from three individual experiments, each performed with at least six repeats; * *p* < 0.05 versus control.

The cells were exposed for 24 h to the IC_50_ concentration of complexes and then changes in the fluidity of cell membranes were measured by fluorescence spectroscopy. It has been shown (Figure 4 and Figure 5) that the tested ruthenium derivatives significantly affect the properties of the plasma membrane of cancer cells, causing changes in its internal viscosity. NALM6 cells were most susceptible to changes in membrane fluidity. For all the used compounds, a statistically significant decrease in membrane fluidity was observed, both in the surface layer (TMA-DPH) and in the hydrophobic core (DAUDA). The level of changes in liquidity depended on the used derivative and amounted to approximately 10–20%. Interestingly, for WM115 cells sensitive to treatment with ruthenium derivatives, changes in membrane fluidity were observed only for the surface layer of the cell membrane. For the remaining cell lines, a 10–15% decrease in membrane fluidity was observed within the hydrophobic core, while tests with TMA-DPH did not show statistically significant changes.

### 2.4. Measurement of Cleaved PARP1 Levels

PARP is a family of enzymes capable of adding branched ADP-ribose chains to proteins. They are activated by the presence of breaks in the DNA strand. These proteins play an important role in DNA repair, and the inhibition of their activity may increase the destruction of cells by cytotoxic drugs. PAPR1 is rapidly recruited into a variety of DNA lesions, where it is involved in the repair of damaged nitrogen bases. PARP inhibitors currently represent a source of great interest in modern chemotherapy; such compounds can potentiate the effects of conventional cytotoxic chemotherapy by inhibiting the DNA repair of cancer cells [19].

**Figure 6:**
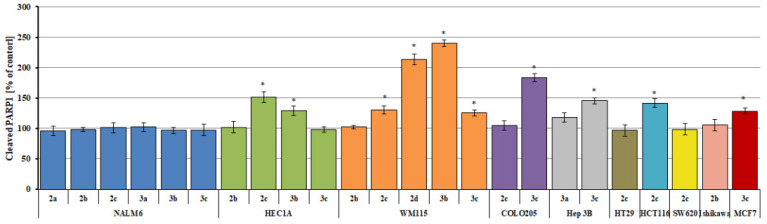
Measurement of PARP1 degradation in NALM6, HEC1A, WM115, COLO 205, Hep 3B, HT29, HCT116, SW620, Ishikawa, and MCF7 treated with investigated compounds. * Results represent mean ± SEM of the data from three individual experiments, untreated control cells arbitrarily taken as 100%, * *p* < 0.05 vs. control.

Compounds **2a**–**3c** (at IC_50_ concentration for individual lines) are able to inhibit PARP1 activity and support its degradation in cancer cells (Figure 6). The results, however, indicate that not all cell lines were sensitive to PARP1 degradation, and protein degradation appears to be strictly dependent on the type of cancer. It is worth noting that the same compound yielded very different levels of PARP1 degradation activity between cancer lines. Interestingly, especially considering the MTT cytotoxicity analysis and the high sensitivity to the tested compounds by the NALM cells, no significant changes in PARP1 degradation were observed for any of the used compounds. The level of degraded PARP1 in NALM6 did not change. No statistically significant changes were observed in HT29, SW620, or Ishikawa cells. In contrast, the greatest changes were observed in WM115 cells exposed to compound **2d** and **3b**, where the level of enzyme degradation increased by approximately 115% and 140%, respectively, compared to controls. An equally high level of protein degradation was observed for compound **3c** and the COLO205 line (approximately 85% increase vs. control). In the case of the remaining cancer lines, depending on the derivative used, PARP1 degradation ranged between 25% and 50%.

### 2.5. Analysis of DNA Damage Using the Alkaline Version of the Comet Assay (Single Cell Electrophoresis)—DNA Comet Assay to Assess DNA Damage of Cancer Cells

**Figure 7:**
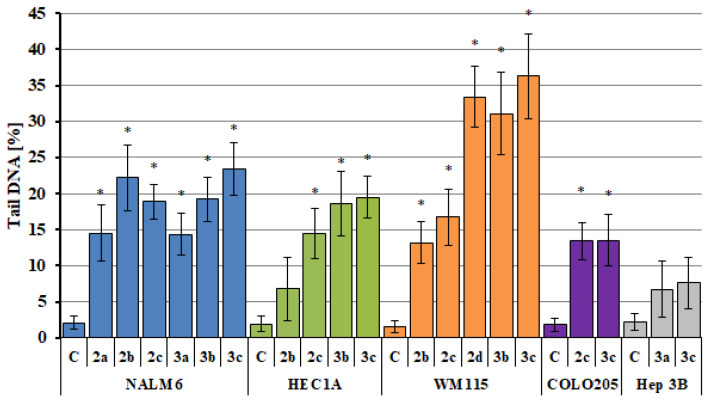

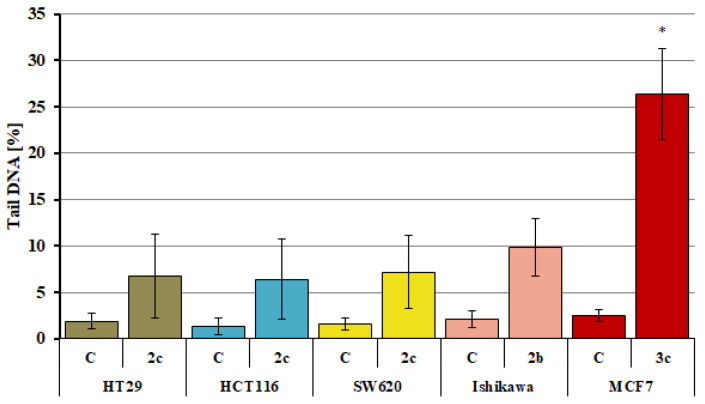
DNA content in the tail of the comet obtained from NALM6, HEC1A, WM115, COLO 205, Hep 3B, HT29, HCT116, SW620, Ishikawa, and MCF7 treated with investigated compounds. The cells were exposed for 24 h to IC_50_ concentration of complexes. C on the axis of the plot represents untreated control cells. * Results represent mean ± SEM of the data from three individual experiments, and for each analysis, 100 randomly chosen comets were counted, * *p* < 0.05 vs. control.

To evaluate the genotoxic properties of the tested ruthenium derivatives, single- and double-strand breaks were identified using the alkaline comet test. Studies have shown (Figure 7) that these compounds have a strong genotoxic effect on the used cancer cell lines. It should be emphasized that the observed changes in cell DNA damage were not always large or statistically significant. No or relatively little DNA damage (about 6–8%) was observed for cancer lines that generally showed greater resistance to the effects of derivatives such as HT29, HCT116, or SW620. Undoubtedly, the results obtained for the WM115, NALM6, or HEC1A lines are worth noting. In the case of WM115, the most effective compounds yielded an increase in DNA damage by as much as 30–35%. Interestingly, in NALM6 leukemia cells, the level of DNA damage was also high (~15–25%) despite no statistically significant degradation of PARP1 polymerase; it is possible that after an extended incubation period without the presence of test compounds, some of this DNA damage would be repaired. Our results indicate, however, that the tested ruthenium derivatives show genotoxic activity against cancer cells. It is likely that the resulting DNA damage may be contributed by ROS, which are generated by ruthenium derivatives and which damage DNA.

**Figure 8:**
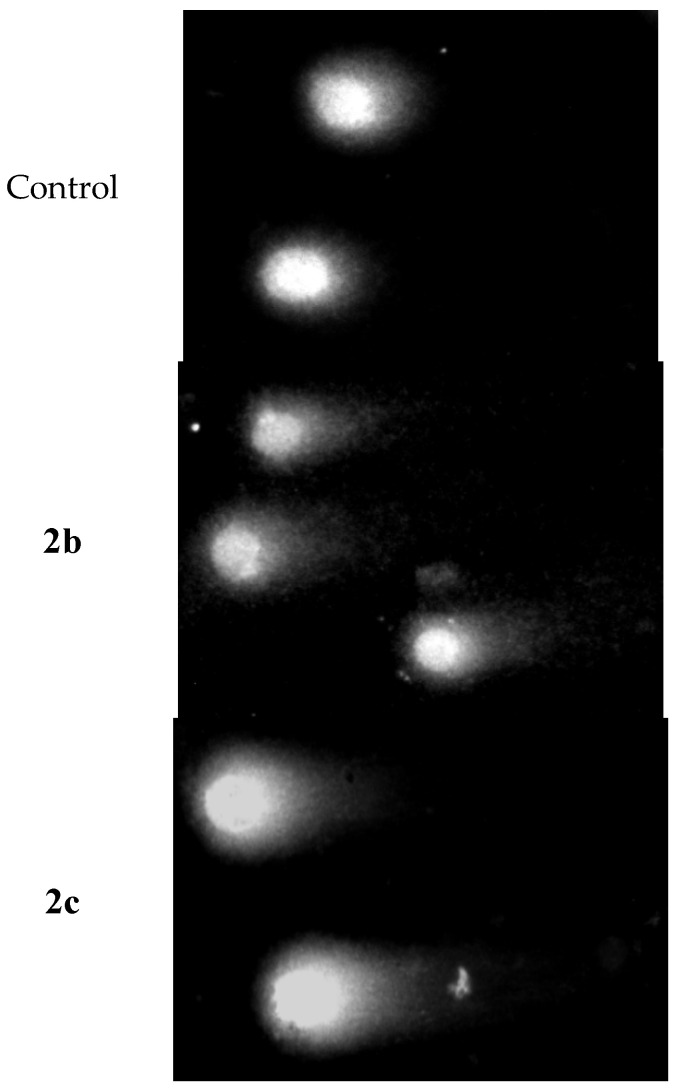

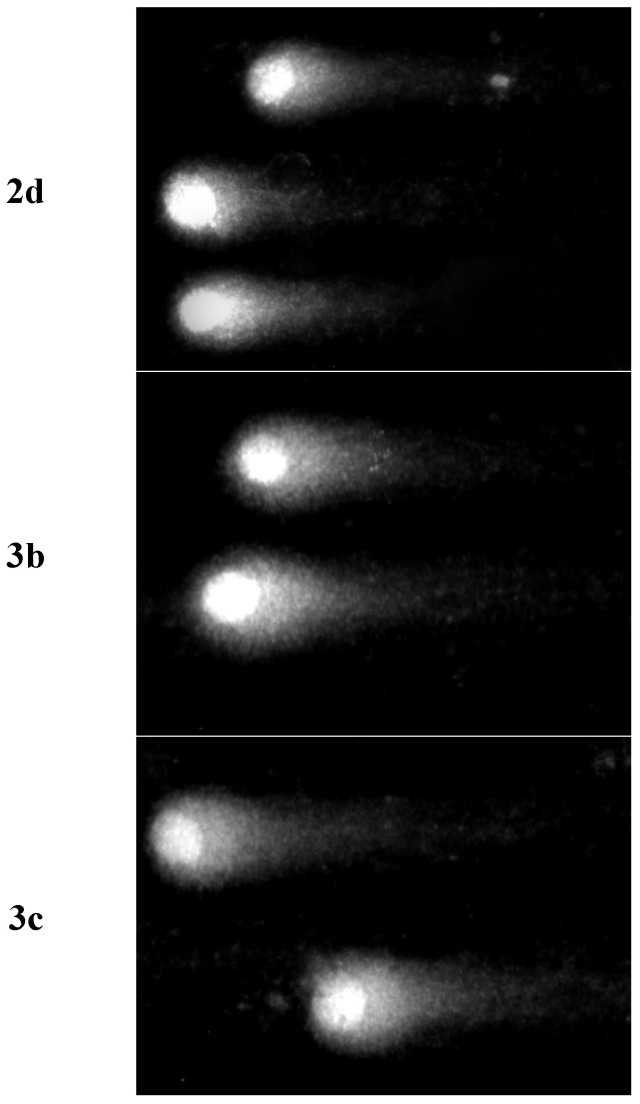
Sample photos of the assessment of DNA damage of cancer cells exposed to ruthenium derivatives. The pictures show WM115 cells treated with ruthenium(II) derivatives **2b**, **2c**, **2d**, **3b**, and **3c**.

Sample images of the DNA damage analysis based on the alkaline comet test (single cell electrophoresis) are shown in Figure 8. Supercoiled DNA loops are visible. They are connected to the nuclear matrix of untreated control cells, which retain a compact comet head-like structure. Staining with the fluorescent dye DAPI (4′, 6-diamidin-2-phenylin) results in the emission of very bright and intense fluorescence. In cells with damaged DNA damage, DNA supercoils are more relaxed and therefore migrate behind the head, creating a comet tail with weaker fluorescence. The cells were incubated with derivatives for 24 h at concentrations of the calculated IC_50_.

### 2.6. Determination of Apoptotic and Necrotic Cell Fractions by Fluorescence Microscopy (Double Staining of Cells with Fluorescent Dyes Hoechst 33258 and Propidium Iodide) 

In addition to the analysis of changes in the mitochondrial potential of cancer cells treated with the tested ruthenium(II) derivatives, a qualitative analysis of apoptosis was performed with the use of two fluorescent dyes: propidium iodide and Hoechst 33258. The method allows for the simultaneous detection of live cells (pale blue fluorescence), those in the early (intense blue fluorescence) and late (violet-blue fluorescence) stage of apoptosis, and necrotic cells (red fluorescence). The treated cancer cells were microscopically evaluated and representative areas were photographed at 150× magnification.

**Figure 9:**
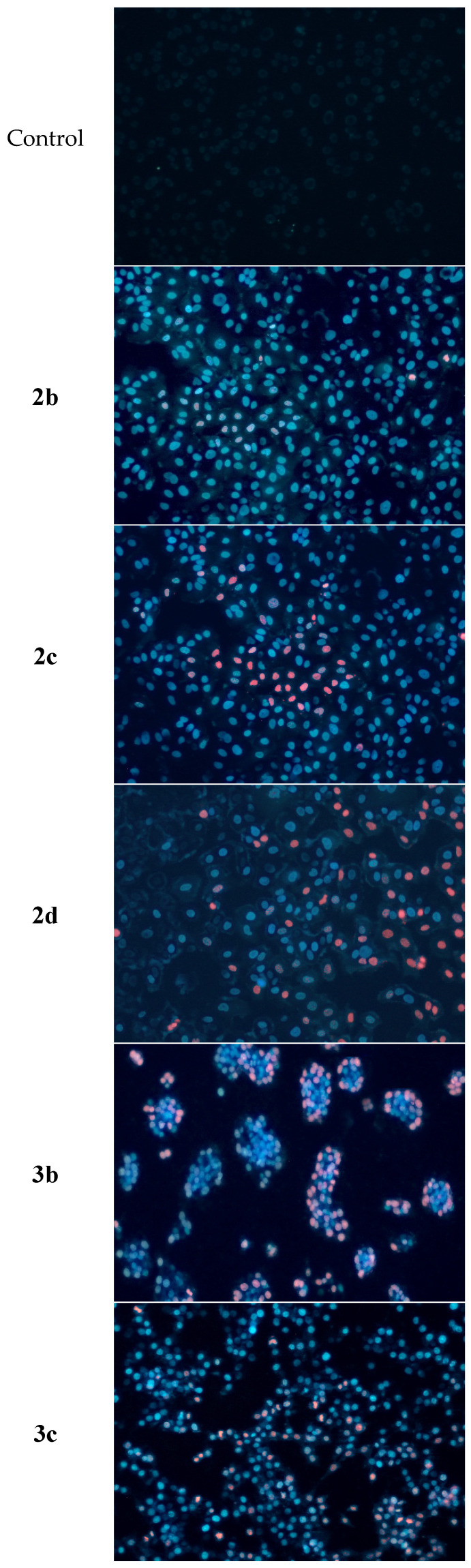
Sample photos of the assessment of cell viability: qualitative assessment of apoptotic and necrotic cell fractions by fluorescence microscopy. The pictures show WM115 cells treated with ruthenium derivatives **2b**, **2c**, **2d**, **3b**, **3c**.

Microscopic analysis showed that ruthenium derivatives induce apoptosis in cancer cells. A significant percentage of cells were found to be in the early phase of apoptosis (intense blue fluorescence). The necrotic cells were still visible; however, they were less clear. Occasionally, a very small percentage of cells was observed in the late phase of apoptosis; however, this may be due to the incubation conditions during the experiment. After 24 h incubation, at concentrations of the calculated IC_50_, the cells were immediately subjected to microscope analysis. Typical fluorescence microscopy images of apoptotic and necrotic cell fractions are shown in Figure 9.

### 2.7. Changes in the Transmembrane Mitochondrial Potential (ΔΨm)

**Figure 10:**
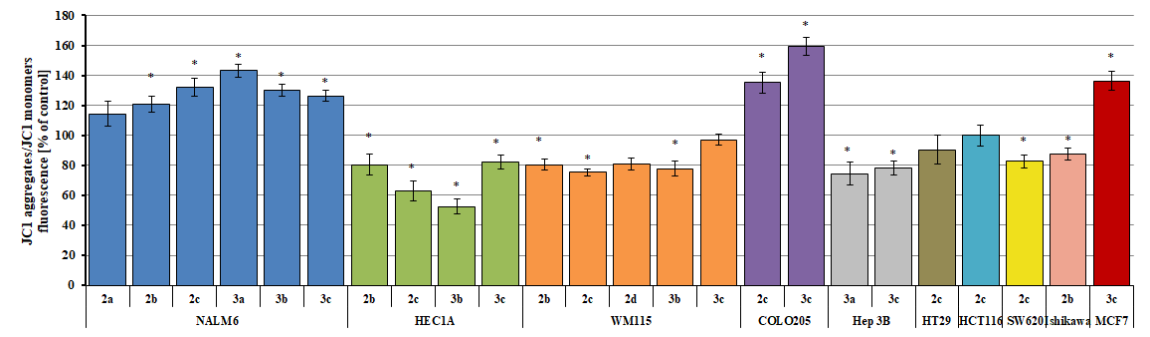
Changes in the mitochondrial potential of NALM6, HEC1A, WM115, COLO 205, Hep 3B, HT29, HCT116, SW620, Ishikawa, and MCF7 treated with investigated compounds. * Results represent mean ± SEM of the data from three individual experiments, and for each analysis, 100 randomly chosen comets were counted, * *p* < 0.05 vs. control.

Changes in mitochondrial potential are seen in the initial stages of apoptosis. They testify to the disturbance of mitochondria and the activation of complex machinery of the programmed cell death pathway. MMP changes were tested using the cationic carbocyanine dye JC-1, which is the most commonly used fluorescent probe in the study of mitochondrial membrane potential changes and the detection of its depolarization/hyperpolarization in cells in vitro. The conducted analysis (Figure 10) showed that the tested derivatives strongly influenced the mitochondrial potential of cancer cells. Only HT29 and HCT116 cells did not demonstrate any statistically significant changes. Interestingly, the compounds, depending on the cell line, caused hyperpolarization or depolarization of mitochondrial membranes. Hyperpolarization of the inner mitochondrial membrane was observed in NALM6, COLO205, and MCF7 cells, where it was approximately 20–60%. The remaining lines demonstrated membrane depolarization, i.e., a decrease in mitochondrial potential. A particularly high decrease (almost 50%) was recorded for HEC1A treated with compounds **2c** and **3b**. In the remaining cases, the decrease in mitochondrial potential was slightly smaller and ranged from ~20%.

### 2.8. Computational Results

The docking scores are collected in Table 4. It should be noticed that, unlike most other docking scoring functions, larger ChemPLP values indicate better ligand binding properties. We have considered two isomeric structures of the ligands: one in which the organic part is interacting with the Ru(II) ion via nitrogen and sulfur atoms, as illustrated by Scheme 1 (labeled S in Table 4), and the other in which the -C(S)NH_2_ moiety is rotated, leading to interaction with Ru(II) ion via two nitrogen atoms (labeled N in Table 4). For these two types of ligands, we have carried out DFT calculations and showed that the former is more stable by over 13 kcal/mol, but as single crystallographic structures of ligands with the PF_6_^−^ counterion were not determined, we have decided to proceed with both isomeric forms of the ligands. As evidenced by the results collected in Table 4, the less energetically favored ligands (N-isomers) also have significantly lower scores, i.e., would bind to targets much more weakly.

**Figure 11:**
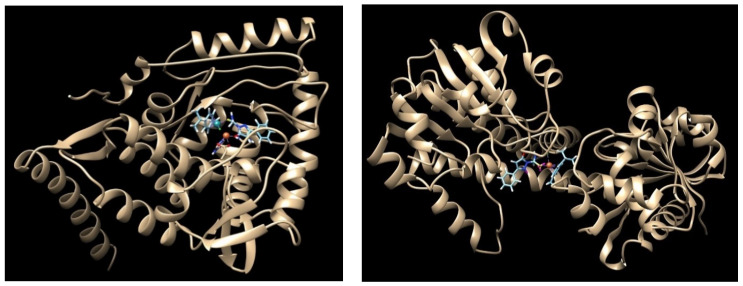

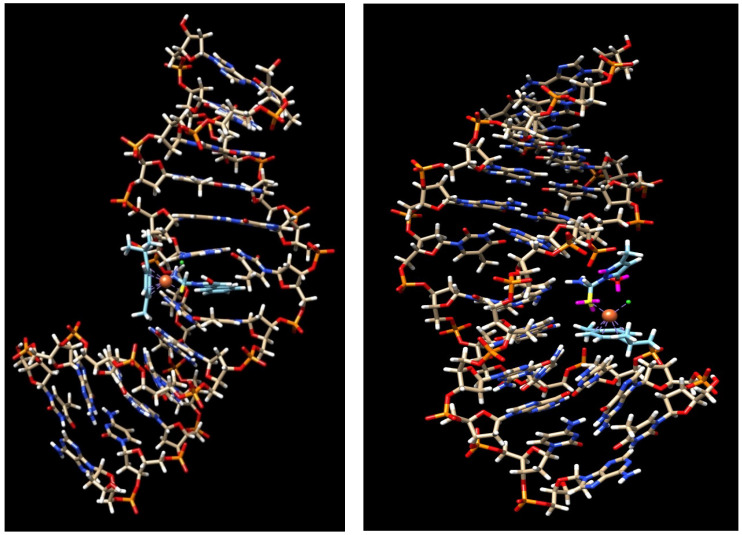
Best poses of S1 bound to 1TOH (**upper left**), 4EDF (**upper right**), and DNA fragment of 1FYY in the intercalation position (**bottom left**) and in the minor groove (**bottom right**).

The S1 (**2c**) ligand exhibits the best binding properties practically in all cases; only in binding to the minor groove is its binding score negligibly worse than that of S2. To illustrate the corresponding orientations, the best poses of S1 (**2c**), i.e., in the active sites of 1TOH and 4EDF, and both binding sites of 1FYY, are given in Figure 11. By comparing the scores across the studied enzymes, we can conclude that the studied compounds generally exhibit the strongest affinity to enzymes, particularly to 4EDF, rather than to DNA strains. The binding of 1FYY exhibited the weakest affinity to the minor groove of 1FYY. 

### 2.9. Lipophilicity Based on RP-TLC

Lipophilicity was determined by RP-TLC, as described preciously [20]. The R_M_ parameters were calculated for the arene–ruthenium(II) complexes **2a**/**3a**–**2d**/**3d** and the ligands **1a**–**1d**.

The lipophilicity appeared to be between 1.64 and 2.59 for compounds **2a**–**2d** and between 2.39 and 2.73 for compounds **3a**–**3d** (Table 5). All the lipophilicity values for complexes **2a**/**3a**–**2d**/**3d** were similar, ranging between 1.5 and 3.

### 2.10. The pH Dependent of Stability of the Complexes

**Figure 12:**
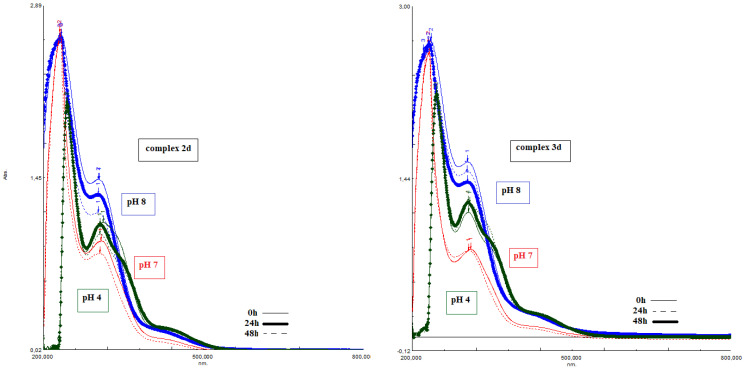
The UV–Vis spectra for complex **2d** (for **left**) and complex **3d** (for **right**) in pH = 4 (green), pH = 7 (red)and pH = 8 (blue) buffer solutions.

The stability under physiological conditions is a crucial part of the process of development for new drugs. Additionally, the leaving groups equip arene–ruthenium(II) complexes with an elegant pH switch and can be protonated at lower pH values, as found in the extracellular matrix of the tumor tissue where they can be cleaved. The formed aqua complex can be highly reactive, leading to the ability to interact with biological targets. This study investigated three different pH values, i.e., 4, 7, and 8. We have chosen these pH values because these represent the physiologically relevant conditions in the human organism. Using this range, pH 4 represents the method of transport of the drug by the stomach, and pH 8 represents the potential accumulation in the duodenum. Spectra were recorded over a time of 24 h and 48 h the range of 220–800 nm. The complexes were studied in 1 × 10^−7^ M solutions. In the earlier article, our team studied the stability of arene–ruthenium(II) complexes in 0.1%DMSO/PBS solution (it corresponded to pH 7). The pH value 7 is the average pH of human blood. The complexes **2a**/**3a**–**2d**/**3d** proved to be stable at neutral pH, as is typically the pH of blood [17]. In this part, we selected for further studies the most promising complex **2d**, which has better activities for the WM-115 cell line than cisplatin. The spectra for this complex show that the first and second maximum absorptions did not change (for pH 4, stable in stomach). In turn, in basic conditions, the second maximum absorption is observed as a loss absorption, without a change in the spectrum (as shown for pH 8). This gives the suggestion that the complex **2d** can partly degrade in the duodenum. The complex **2d** can be promising for the optimal bioaccumulation of the drug in the blood. The complex **3d** presented similar stability at various pH conditions, Figure 12.

## 3. Discussion

The present study evaluates the anticancer activity of arene–ruthenium(II) derivatives **2a**/**3a**–**2d**/**3d** for selected human cancer lines. The procedures fall within the scope of oriented basic research, and are aimed at gaining new knowledge in anticancer activity profile assessment.

The article focuses on arene–ruthenium(II) complexes, a group of compounds that exhibit both antibacterial and antitumor activity [21]. One of the most promising classes of these compounds are the so-called RAPTA compounds based on the arene-Ru(II) unit linked to amine moieties. RAPTA comprises a large group of compounds which have been extensively investigated for their tumor-inhibiting properties. In vivo studies have shown some RAPTA complexes to demonstrate excellent inhibition of metastatic properties. Sadler and co-workers report that the typical half-sandwich “piano-stool” structure of ruthenium complexes seems to be advantageous for anticancer activity. The more hydrophobic an arene-ligand and a single ligand exchange site are, the more associated they are with high cytotoxicity. When the ethylenodiamine ligand is replaced with acetylacetonate, the cym and bip complexes are much more cytotoxic than the dihydroanthracene complex. Organometallic “piano-stool” complexes in general contain a range of various features which are very useful in drug design [22].

The search for an effective alternative to existing anticancer drugs is a great challenge for researchers. The eight arene–ruthenium(II) complexes containing carbothioamidopyrazoles as ligands were tested against fifteen adherent and one suspension human cancer cell lines. Our present findings show that the anticancer activity of arene–ruthenium(II) complexes **2a**/**3a**–**2d**/**3d** is influenced by changes in structure, the presence of carbonyl, hydroxyl, phenyl groups in the pyrazole ligand, or a change in the counter ion. All complexes demonstrated much better anticancer activity against the selected cell lines than their corresponding ligands. It was shown that the alkyl substituents (methyl in **2a**/**3a** and ethyl groups in **2b**/**3b**) in the C-3 and C-5 positions of the carbothioamido pyrazole of the tested *p*-cymene-ruthenium(II) complexes did not cause an increase in anticancer activity. In the pyrazole ring of the **2c**/**3c** complexes, a satisfactory effect was observed for the phenyl group in the C-3 position and the hydroxyl group in the C-5 position. In turn, in the pyrazole ring of the **2d** complex, the methyl group in the C-3 position and hydroxyl group in the C-5 position gave satisfactory results for WM-115 cell lines; i.e., better than cisplatin. The use of the anions [Cl]^−^ and [PF_6_]^−^ did not have a strong influence on this effect but could increase solubility. It is also worth noting that the described complexes in question are stable in the water/DMSO solution, as described previously [17]. The complex **2d** can be promising for the optimal bioaccumulation of the drug in the human organism.

The following seven compounds turned out to be most active against the tested cancer cell lines: **2a**, **2b**, **2c**, **2d**, **3a**, **3b**, and **3c**. Very attractive results were obtained for WM115 cells, where **2d** showed much better biological activity than the reference compounds cisplatin [16] and NAMI-A. It should be emphasized that cisplatin is very often used as a gold standard reference compound in cytotoxic studies on new metal compounds. In research on the development of new, potential chemotherapeutic agents, the aim is to achieve better properties of cisplatin derivatives while lowering systemic toxicity. NAMI-A is much less cytotoxic than cisplatin and has a different mechanism of biological action. Unlike cisplatin, NAMI-A has been shown not to inhibit primary tumor growth and is not highly cytotoxic to cancer cells. However, it significantly reduces the rate at which metastases occur [11]. By using NAMI-A as a reference compound, we wanted to relate our results to a commercially available compound containing ruthenium in its structure. However, we emphasize that the cytotoxicity results of the analyzed derivatives should be related primarily to cisplatin.

In addition to such attractive anticancer properties, this compound demonstrated very good selectivity in its biological action. Our studies using HMEC-1 normal endothelial cells indicate that the **2d** complex is an attractive chemotherapeutic agent. The IC_50_ concentration calculated for **2d** against HMEC-1 was 163 µM, clearly indicating low systemic toxicity. Based on these preliminary results, we can assume that, after more detailed biological analyses, this compound may represent an attractive chemotherapeutic agent in itself, or serve as a starting point for the synthesis of further, even more biologically-active compounds in the treatment of skin cancer.

The tested derivatives demonstrated different degrees of biological activity with regard to the remaining lines of cancer cells. Their IC_50_ concentration was often lower than that of cisplatin. Importantly, however, significantly higher anticancer activity was observed for all tested ruthenium derivatives compared to NAMI-A, indicating the high potential of these derivatives as a potential new class of chemotherapeutic agents. Among the complexes, selected on the basis of their cytotoxicity and calculated IC_50_ concentrations, compounds **2c** and **3c** undoubtedly deserve more attention. They were characterized by the broadest spectrum of biological anticancer activity against all studied cancer cell lines. Both **2c** and **3c** displayed low IC_50_ values (10–50 µM) against seven tumor lines (HT29, Colo205, SW620, LoVo, HEC1A, WM115, and NALM6 for **2c**, and Colo205, LoVo, Hep3b, HEC1A, MCF7, WM115, and NALM6 for **3c**). These compounds generated oxidative stress in cancer cells, activated the apoptosis pathway, and induced DNA damage, which was associated with the inhibition of PARP1 polymerase activity.

Several previous studies on arene–ruthenium (II) complexes incorporated with different ligands have shown very good biological activity towards various cancer cell lines [23,24,25,26]. However, the cytotoxic properties of the analyzed ruthenium derivatives do not provide any clear indication of whether arene–ruthenium(II) complexes containing [Cl]^−^ (**2a**–**d**) show better anticancer properties than those containing [PF_6_]^−^ (**3a**–**d**). Therefore, based on our basic research, it is not possible to clearly indicate a better ligand for ruthenium derivatives in the context of their anticancer activity. One difference was observed regarding cytotoxic properties (MTT test): the ruthenium derivatives with [Cl]^−^ achieved an IC_50_ below 50 µM for seven of the fifteen tumor lines (**2a**–**2d**) while those containing [PF_6_]^−^ (**3a**–**3d**) achieved it for six. The results obtained for compound **2d** against the WM115 line are particularly interesting and promising; however, compound **3d** significantly reduced the biological activity (IC_50_ > 200 µM).

Interestingly, a different tendency was observed for **2c** and **3c**. Derivative **2c** was characterized by high cytotoxic activity, with which IC_50_ > 50 µM was observed for seven cell lines: HT29, Colo205, SW620, LoVo, HEC1A, WM115, and NALM6. Similarly, **3c** achieved IC_50_ > 50 µM against six cell lines: Colo205, LoVo, HEC1A, MCF-7, WM115, and NALM6. Comparing this pair of **2c**–**3c** derivatives, characterized by the broadest spectrum of biological activity against the studied types, **2c** demonstrated slightly better cytotoxic activity. However, we would like to emphasize that the differences in cytotoxic activity observed between the compounds tested in this work probably depend on both their structure and the individual characteristics of the target cancer cells. This hypothesis is also confirmed by the subsequent molecular studies of the activity of the tested ruthenium derivatives.

In addition to the high cytotoxic activity of potential chemotherapeutic agents against cancer cells, their appropriate selectivity and the associated low systemic toxicity are significant challenges. Commercially used drugs also kill normal cells at similar concentrations to cancer cells. The consequence of this limited selectivity is that patients cannot receive the drug doses needed to kill all the cancer cells. Helpful in assessing the selectivity of a given compound is the selectivity index (SI) parameter, characterized as the ratio of the toxic concentration of the sample towards cancer cells to its effective bioactive concentration (relative to normal cells). The ideal drug can kill cancer cells, but it should not affect normal cells, i.e., have a high SI [27,28,29]. Based on this information and the results obtained by us for the tested compounds, it can be confidently stated that they are characterized by a high selectivity index. The results of MTT cytotoxicity for HMEC-1 cells indicate a very high specificity and selectivity of the action of the analyzed ruthenium derivatives. For the most active ruthenium(II) complexes, *viz.* **2a**, **2b**, **2c**, **2d**, **3a**, **3b**, and **3c**, the IC_50_ value for HMEC-1 was significantly higher (about 3–5 times) than for cancer cells, ranging between 125 and 200 µM. This represents exceptionally high selectivity by ruthenium(II) complexes towards normal and cancer cells. Undoubtedly, this feature is extremely desirable from the point of view of the potential future uses of *p*-cymeneruthenium(II) complexes as potential chemotherapeutic agents. Molecular studies on the effect of derivatives on ROS generation, apoptosis induction, and the formation of complexes with DNA and PARP-1 enzyme have been used to determine the anticancer properties of the studied ruthenium(II) complexes.

The transport of substances into cells has a crucial influence on the biological activity of any drug. The trail along which molecules pass membranes or other barriers can be traced by specific indicators such as drug lipophilicity. Generally, more lipophilic molecules penetrate the cell membrane more easily because they interact better with the fatty acids of the bilayers; however, in compounds which are more lipophilic, the molecules will stick to the lipid bilayers and will not penetrate the membrane. The lipophilicity can be described as logP, which is a decadic logarithm of the equilibrium concentration ratio of a compound in two immiscible phases. Generally, an *n*-octanol/water system is a suitable model to project an environment for drug transport in a living organism, as *n*-octanol possesses a similar polarity to lipid bilayers [30,31]. The discussed compounds **2a**/**3a**–**2d**/**3d** presented acceptable lipophilicity, which allows for bioaccumulation in biological systems. According to Lipinski’s rule and drug-likeliness parameters, the obtained results give hope for finding candidates in medicine which will be as good as existing drugs.

One basic mechanism used by metal complexes, including ruthenium(II) derivatives, to induce apoptosis is ROS production [32]. ROS production often results in the dysfunction of basic cell organelles, and, consequently, the activation of apoptosis/autophagy pathways [32,33,34]. Our research is in line with previous reports of the induction of oxidative stress and ROS in cancer cells by arene–ruthenium(II) complexes (**2a**/**3a**–**2d**/**3d**) [35,36]. Mitochondria participate in ROS-induced oxidative stress, resulting in the loss of membrane potential and the activation of cell death as part of the intrinsic apoptotic pathway. The analyzed ruthenium derivatives significantly increased the level of ROS in cancer cells, as detected by the DHET probe (preferably detects superoxide O_2_^•−^) and the H_2_DCFDA probe (detection of hydrogen peroxide H_2_O_2_). Compounds **2a**–**2d** favored O_2_^•−^ generation in tumor cells, whereas **3a**–**3d** favored H_2_O_2_. However, it is important to emphasize that for the HEC1A and WM115 lines, no statistically significant changes in superoxide O_2_^•−^ were observed after exposure to any test compound; in these cell lines, ruthenium derivative-dependent oxidative stress was conditioned by the formation of hydrogen peroxide. In addition, it is also worth noting that the level of RNS increased in some cases. In turn, the activation of free radical reactions in cancer cells was correlated with the damage to their cell structures.

One of the main events of ROS-induced cell death is the loss of cell membrane integrity and membrane lipid peroxidation [35]. After exposure, changes in membrane fluidity were observed in the deeper layers of the hydrophobic tails (DAUDA probe) and on the surface of the hydrophilic heads of the lipid bilayer (TMA-DPH). Interestingly, compounds **3a**–**3d** showed slightly better activity in the hydrophobic region, while compounds **2a**–**2d** caused strong peroxidation within the hydrophilic part. In addition, it should be emphasized that for the WM115 and HEC1A lines, significant changes in fluidity were visible only with the use of TMA-DPH, while for cancer cell lines HT29, HCT116, and SW620, fluidity changes were observed only in the deeper layers (DAUDA). As in the case of cytotoxicity, it is difficult to indicate which of the complexes, either those with chloride ions (**2a**–**3d**) or those with hexafluorophosphoric ions (**3a**–**3d**), increases the anticancer activity of the tested derivatives. The observed differences in biological activity may have resulted from differences in the structure of ruthenium complexes and in individual tumor types. Our findings on generating oxidative stress by ruthenium derivatives are consistent with previous studies. Several previous reports indicate the pro-oxidative properties of ruthenium derivatives [37,38,39]. Moreover, as the results show, the generation of ROS in cancer cells dependent on ruthenium derivatives plays a crucial role in shaping the total anti-cancer potential of these compounds. Vyas et al. [39] indicate that arene–ruthenium(II)-tetrazole compounds induced ROS in cancer cells. Notably, as the authors indicate, the induction of oxidative stress and the formation of ROS were mediators of the initiation of apoptosis of cancer cells. Similar results were also obtained for NHC-coordinated ruthenium(II)-arene complexes, and pyrazole appended quinoline-BODIPY-based arene–ruthenium complexes, where the authors observed a significant increase in ROS in cancer cells leading to their death by apoptosis, especially in the mitochondrial (internal) pathway [37,38,39].

The pro-apoptotic properties of the ruthenium derivatives were confirmed by the observed depolarization of the mitochondrial membranes in cancer cells. In addition, the activation of the programmed death pathway was indicated by microscope analysis using Hoechst/propidium iodide fluorescent dyes [33,34]. The analysis of changes in the mitochondrial potential of cancer cells and the use of fluorescent dyes and microscopy allowed for a qualitative assessment of apoptosis induction. The pro-apoptotic properties of arene–ruthenium (II) complexes have been confirmed in a number of recent studies [40,41,42,43]. The apoptosis-promoting effect on human cancer cells takes place primarily via the internal pathway with the participation of mitochondria; this is confirmed by our present findings, *viz.* the depolarization and hyperpolarization of mitochondrial membranes, fluorescence microscopy, and the presented photos after staining with propidium iodide and Hoechst.

Undoubtedly, a valuable addition to our research in the future will be the planned analysis of gene expression and the protein products involved in apoptosis, especially Bcl-2 family proteins and caspases. As indicated by earlier reports, ruthenium derivatives activate pro-apoptotic proteins of the Bcl-2 family (e.g., Bax) in cancer cells with high efficiency, while reducing the expression of anti-apoptotic Bcl-2. Moreover, programmed cell death is preceded by the ruthenium derivative-dependent activation of caspases [43,44,45]. In this context, it is reasonable to expand molecular biology research using arene–ruthenium(II) complexes with carbothioamidopyrazoles studied by us.

Our genotoxicity studies of the analyzed ruthenium derivatives are in line with previous reports. The arene–ruthenium(II) complexes are known to damage the DNA of cancer cells [30,31,41,42]. A particularly interesting finding concerns the interaction between arene–ruthenium(II) complexes and PARP1 polymerase, especially regarding their anticancer properties. Very few, if any, studies have examined this topic to date. Pavlovic et al. [31] report that ruthenium(II)-arene complexes bearing benzamide derivatives could effectively inhibit PARP1 activity. Additionally, in the cases of studies using arene-ruthenium(II) acylpyrazolonato complexes, PARP degradation was observed with the participation of caspases activated by the tested derivatives [43]. The inhibition or degradation of key DNA repair enzymes such as PARP-1 is often associated with genotoxicity [25,46,47]. Our findings confirm that *p*-cymene–ruthenium(II) complexes have genotoxic properties. Most of the analyzed compounds caused a high (about 15–30%) increase in DNA damage in the tested cancer cells. However, it is impossible to indicate which complexes **2a**–**2d** or **3a**–**3d** conditioned the better genotoxic properties of the analyzed ruthenium derivatives; both compounds **2a**–**2d** and **3a**–**3d** yielded similar findings. Interestingly, once again, the intestinal cancer lines HT29, HCT116, and SW620 (but not COLO205) and the Hep3B liver cancer cells did not suffer DNA damage after exposure to the tested compounds. Significant DNA damage was recorded in the remaining cells, especially for the WM115 line. It is also worth noting that the analyzed ruthenium(II) derivatives caused the degradation of PARP-1, involved in the repair of DNA damage. The ability of arene–ruthenium(II) derivatives to interact with PARP-1 was also confirmed both in silico and in vitro. In silico docking indicates a high affinity of ruthenium(II) complexes for PARP-1 polymerase. In addition, ELISA analysis clearly emphasizes that these compounds cause protein degradation.

In silico molecular docking results confirm the high affinity of ruthenium derivatives to PARP1 polymerase and their ability to inhibit it. It was found that **2c** complexes exhibit the best binding properties in nearly all cases. These results are reflected in in vitro studies using human cancer cells, but not directly. In the in vitro studies, compound **2c** showed high PARP1 inhibition activity, which was the highest among all tested ruthenium derivatives in most cases. For example, PARP1 was significantly more degraded after incubation with compounds **2d** and **3b** in WM115, but not by compound **2c** in NALM 6 cells. A comparison of the results of the in silico and in vitro analysis of PARP1 inhibition therefore suggests that additional mechanisms are involved in the interaction between ruthenium(II) derivatives and the polymerase. Our findings indicate that the level of PARP1 degradation probably depends inter alia on the type of cancer cell and its individual characteristics, and its expression of individual genes or protein activity. It should also be emphasized that complex **2c** was characterized by the broadest spectrum of anticancer activity. This compound was highly active against of seven out of the ten cancer cell lines used in all experiments. It is possible that the ability of **2c** to interact with PARP1 is one of the key mechanisms shaping its total cytotoxic and anticancer potential. The results of PARP degradation analysis obtained by us undoubtedly indicate a high pro-apoptotic potential of the studied ruthenium derivatives. The degradation of this enzyme is one of the key steps in the apoptosis process and occurs through the activation of cysteine proteases (caspases) [43].

An important issue in the case of the analyzed ruthenium derivatives is their fate and distribution in cancer cells; as studies using ruthenium derivatives indicate, this can be diverse. Unlike cisplatin, ruthenium compounds are localized primarily within the lipid bilayer of the cell membrane and do not cross the barrier of the cell nucleus [48]. Of course, a direct interaction between ruthenium compounds and DNA is possible [49,50]; however, such a mechanism is closely related to the structure of the compound, and different cellular fates have been noted for KP1019 and NAMI-A [51].

Regarding our research and ruthenium complexes, it is not easy to clearly indicate their cellular location and main site of action. Firstly, the molecular biological activity and lipophilicity data suggest that the main location of the tested ruthenium derivatives is the area between the lipid bilayer of the cell membrane, and that it is probably from this cellular location that the compounds exert their anticancer activities via cell signaling. The ROS complexes generated by the complexes within the lipid bilayer may lead to direct lipid peroxidation and the activation of apoptosis signaling pathways. Moreover, it is also possible that the analyzed compounds may indirectly induce DNA damage via, e.g., ROC generation. It is worth noting that genotoxicity may not always be directly related to the entry of compounds into the cell nucleus. However, to determine the true cellular fate of the analyzed ruthenium complexes, it is necessary to analyze the distribution of these compounds in cancer cells, and this is intended for a future study. Indeed, the cellular fate of these complexes may vary between different types of cancers.

## 4. Experimental Section, Material and Methods

The arene–ruthenium(II) complexes **2a**/**3a**–**2d**/**3d** and their appropriate ligands **1a**–**1d** were prepared according to the procedures described in our literature [16,17].

### 4.1. Cell Lines and Cell Culture

Cytotoxicity was evaluated against fifteen adherent human cancer cell lines using the MTT test: colorectal adenocarcinoma cells Caco2 (ATCC^®^ HTB-37™), colorectal adenocarcinoma cells HT29 (ATCC^®^ HTB-38™), colon colorectal carcinoma HCT116 (ATCC^®^ CCL-247™), Caucasian colon adenocarcinoma COLO205 (ATCC^®^ CCL-222™), colorectal adenocarcinoma SW620 (ATCC^®^ CCL-227™), colorectal adenocarcinoma grade IV LoVo (ATCC^®^ CCL-229™), hepatocellular carcinoma Hep 3b (ATCC^®^ HB-8064™), endometrium adenocarcinoma HEC1A (ATCC^®^ HTB-112™), Ishikawa (99040201; Sigma–Aldrich Sigma, St. Louis, MO, USA), cervical adenocarcinoma cells HeLa (ATCC^®^ CCL-2™), lung epithelial carcinoma A549 (ATCC^®^ CRM-CCL-185™), breast epithelial adenocarcinoma cell positive for estrogen and progesterone receptors MCF7 (ATCC^®^ HTB-22™) and negative for expression of estrogen receptor HCC38 (ATCC^®^ CRL-2314™), acute promyelocytic leukemia HL-60 (ATCC^®^ CCL-240™), skin melanoma WM115 (ATCC^®^ CRL-1676™), and 1 suspension cancer cell line: acute lymphoblastic leukemia NALM6 (purchased from the German Collection of Microorganisms and Cell Cultures). HT29, NALM6, HCC-38 and HL-60 cells were cultured in RPMI 1640 medium (Thermo Fisher Scientific, Waltham, MA, USA) supplemented with 10% fetal bovine serum and antibiotic (gentamicin 25 μg ml^−1^) (Gibco, Grand Island, New York, NY, USA). For the remaining tumor cell lines, Dulbecco’s minimal essential medium (DMEM, Lonza, Visp, Switzerland) supplemented with 10% fetal bovine serum and antibiotic instead of RPMI 1640 was used. In addition, Human Microvascular Endothelial Cells (HMEC1), which were obtained from the American Type Culture Collection (Manassas, VA, USA), were used as a model of normal cells. HMEC-1 were cultured in MCDB131 medium (Corning Life Sciences, Corning, NY, USA), supplemented with 10% fetal bovine serum, 10 ng/mL Epidermal Growth Factor (Millipore, Burlington, MA, USA) and 10 mM glutamine (Corning Life Sciences). All cells were grown at 37 °C in humidified atmosphere of 5% CO_2_ in air. The exponential growth of cells was maintained by regular passaging at 90% confluence three times a week using 0.025% trypsin/EDTA (Gibco, Grand Island, New York, NY, USA). 

### 4.2. Cytotoxicity Assay

The cytotoxicity was tested by a standard MTT [3-(4,5-dimethylthiazol-2-yl)-2,5-diphenyltetrazolium bromide] (Sigma, St. Louis, MO, USA) microplate cell viability assay [21]. The anticancer activity of the investigated compounds was evaluated in vitro on the basis of their ability to inhibit the proliferation of various cancer cells. Exponentially growing cells were seeded a day before the experiment into a 96-well microplate (Nunc, Roskilde, Denmark) at a density up to 6–8 × 10^3^ cells/mL (depending on the cell line) and exposed to the test compounds at a wide range of concentrations for 48 h. Subsequently, various concentrations of the studied compounds (10^−7^–10^−5^ M) freshly prepared in DMSO (final concentration of <0.1% DMSO) and diluted with complete culture medium were added. After 48 h incubation, the cells were immediately subjected to a cell viability assay. At this time point, the number of viable cells in each well was estimated by the MTT test. Briefly, 50 µL (5 mg/mL final concentration) of MTT was added to each microplate well. This procedure was then followed by a three- to four-hour incubation at 37 °C. After incubation, MTT solution was replaced with 100 µL DMSO/well, which solubilized the formazan crystals formed by the dehydrogenases of metabolically active cells. Purple formazan absorbance was measured spectrophotometrically at 570 nm. Cytotoxicity was evaluated on the basis of their IC_50_ concentrations (µM), i.e., those that reduced cell viability by 50% compared to untreated control cells. The two reference compounds that were used in the cytotoxicity study, cisplatin and NAMI-A, were also kept under the same incubation conditions. 

### 4.3. Measurement of Membrane Fluidity

Any changes in membrane fluidity and the regions in which these changes occurred were identified by changes in the fluorescence anisotropy of two fluorescent probes 1-(4-(trimethylamino) phenyl)-6-phenylhexa-1,3,5-triene (TMA-DPH, Cayman Chemical Company, East Ellsworth Road Ann Arbor, MI, USA) and 11-(Dansylamino)undecanoic acid (DAUDA; Sigma, St. Louis, MO, USA). DAUDA was located in the hydrophobic region of the lipid membrane, while the TMA-DPH probe was near the hydrophilic part of the lipid bilayer. Greater fluorescence anisotropy indicates decreased membrane fluidity [52]. The final concentration of fluorescent labels in solution was 1 µM. The cells were seeded into a 6-well plate 24 h before the treatment. After this time, the investigated compounds were added at their IC_50_ concentrations. After 24 h of incubation, the cells were trypsinized and moved into Eppendorf tubes (0.5 mL cells suspension/sample). The cell pellet was washed twice with cold (0–4 °C) PBS. In the next step, the PBS was removed and a cold (0–4 °C) Tris-HCl/KCl buffer (50 mM Tris-HCl, 0.15 M KCl) was added to the cell pellet, and samples were placed on ice. A fluorescent probe was added to the samples, which were incubated on ice in darkness. Immediately afterwards, the samples were subjected to 10 min of incubation under culture conditions, following which, the mobility of the probes in the lipid membrane was analyzed at subsequent excitation/emission wavelengths: 355/516 nm for DAUDA and 355/430 nm for TMA-DPH. Fluorescence anisotropy was measured with a Cary Eclipse fluorescence spectrophotometer (Varian, Inc., Palo Alto, CA, USA).

### 4.4. Measurement of Reactive Oxygen and Nitrogen Species

The measurement of intracellular reactive oxygen (ROS) and nitrogen (RNS) species production in endothelial cells was recorded by changes in the fluorescence of three different fluorescent probes: 2′,7′-dichlorofluorescin diacetate (H_2_DCFDA; Sigma-Aldrich, St. Louis, MO, USA) and dihydroethidium (DHEt; Cayman Chemical Company, MI, USA). These are fluorescent probes which may react with several reactive oxygen species. The cell-permeant H_2_DCFDA passively diffuses into cells and is retained on the intracellular level after cleavage by intracellular esterases. Upon oxidation by ROS (including hydrogen peroxide, hydroxyl radicals, and peroxynitrite), the nonfluorescent H_2_DCFDA is converted to a highly-fluorescent green product—2′,7′-dichlorofluorescein (DCF) (absorption/emission: 504/529 nm) [53]. Dihydroethidium (DHEt) also possesses the ability to freely permeate cell membranes and is mainly used to monitor superoxide production. Dihydroethidium itself shows blue fluorescence (absorption/emission: 370/420 nm) but in the cell cytoplasm, it is oxidized by superoxide to 2-hydroxyethidium and becomes red (absorption/emission: 535/610 nm) upon DNA intercalation [54]. Diaminofluorescein-FM (DAF-FM Diacetate, Invitrogen, Carlsbad, CA, USA) is a cell permeant reagent commonly used to detect and quantify even low concentrations of RNS, especially nitric oxide (NO). DAF-FM is essentially nonfluorescent until it reacts with NO, whereupon it forms fluorescent benzotriazole (absorption/emission: 495/515 nm) [55]. The final concentration of fluorescent labels in solution was 5 µM. Fluorescence intensity was measured with a Cary Eclipse fluorescence spectrophotometer (Varian, Inc.) after 30 min of incubation under culture conditions at listed excitation/emission wavelengths: 504/529 nm for H_2_DCFDA, 535/610 nm for DHEt, and 495/515 nm for DAF-FM. The cells were seeded in black 96-well fluorometric microplates at a density of 10 × 10^3^ cells per well 24 h before the treatment. The cells were incubated with the investigated compounds (IC_50_ concentration) for 24 h. After incubation, the medium was removed and 50mL of HBSS buffer containing H_2_DCF-DA or DHEt was added to each well. The cells were incubated with the fluorescent probes for 30 min in a CO_2_ incubator, in darkness at 37 °C, and the DCF/DHEt fluorescence was measured.

A similar procedure was used for RNS. Monolayers of treated and control cells were seeded into black 96-well fluorometric microplates. After incubation with the investigated compounds (IC_50_ concentration), the medium was removed and 50mL of HBSS buffer containing DAF-FM (final concentration 5 µM) was added to each well. The cells were incubated with a fluorescent probe for 30 min in a CO_2_ incubator, in darkness at 37 °C. After incubation, the probe was removed and replaced with fresh HBSS buffer, and then incubated for an additional 30 min to allow for complete de-esterification of the intra cellular diacetates. Fluorescence excitation and emission maxima are 495 and 515 nm, respectively.

### 4.5. Measurement of Changes in Mitochondrial Potential (*Δ*Ψm) Using the Microplate Spectrofluorimetric Method with the JC1 Fluorescent Probe

JC1 is a cationic carbocyanine dye widely used as a fluorescent probe to detect mitochondrial membrane depolarization. Mitochondria demonstrate selective accumulation of JC-1 that is highly dependent on the transmembrane mitochondrial potential (Ψm) [56,57]. In normal cells, the appropriate mitochondrial membrane potential (MMP) ensures the efficient functioning of energy metabolic pathways. Under normal conditions, cells have a high mitochondrial membrane potential (MMP), estimated at −120 to −180 mV and conditioned by the appropriate distribution of protons (H^+^). In pathological conditions, there is a sharp decrease in ATP production, which causes energy depletion and the depolarization of MMPs. Changes in the mitochondrial potential are characteristic of initial stages of apoptosis.

Changes in the MMP can be detected by the use of the JC1 fluorescent probe. JC1 accumulates in large amounts in a hyperpolarized mitochondrial membrane (−140 mV), where it forms aggregates emitting red fluorescence (λ_em_ = 590 nm). During depolarization (MMP −100 mV) and permeation of the mitochondrial membrane, JC-1 concentration decreases and aggregates break down into monomers emitting green fluorescence, similar to that of fluorescein (λ_em_ = 525 nm). The transition of JC1 from the aggregated to monomeric form is associated with shifts in its fluorescence spectrum and with a change from orange to green fluorescence. These features make JC1 a sensitive marker of MMP changes, and measuring the aggregate to the monomer ratio of fluorescence a convenient and reliable method for assessing MMP changes in cells [58]. The ratio of fluorescence of aggregates (λ_em_ = 590 nm) and monomers (λ_em_ = 525 nm), i.e., 590 nm/525 nm, reflects the level of damage to the mitochondrial cellular membrane.

Cells (10 × 10^3^/well) were seeded in black 96-well plates and grown for 24 h to reach the log phase. After 24 h of cell culture, the test compounds were added at the IC_50_ concentration and incubated for three hours in a CO_2_ incubator at 37 °C. After removing the medium, the cell monolayer was washed twice with PBS solution and 50 μL of the JC-1 probe at a final concentration of 5 μM was added to the wells of the plate. The cells were incubated with the dye for 30 min in a CO_2_ incubator. Subsequently, the changes in the fluorescence of monomers (λ_ex_ = 485 nm and λ_em_ = 525 nm) and aggregates (λ_ex_ = 530 nm and λ_em_ = 590 nm) using JC-1 were monitored. The results of the tested trials were presented as the ratio of fluorescence of aggregates and JC1 monomers as a percentage of control values, which was assumed to be 100%.

### 4.6. Analysis of DNA Damage Using the Comet Method in the Alkaline Version (Single Cell Electrophoresis)

Single-cell gel electrophoresis, also known as the comet test, is a fast and sensitive method for assessing DNA damage at the single-cell level. This method is used for the detection of genotoxic and carcinogenic agents. Cells incubated with the test compound are plated on agarose gel, spread on a microscope slide, and then lysed and electrophoresed. Under alkaline conditions, both single-stranded and double-stranded cracks are detected. The released DNA denatures and moves towards the anode on agarose gel during electrophoresis. The image, obtained after staining with the DAPI fluorescent dye, resembles a “comet” with a distinct “head” (intact DNA) and “tail “(damaged DNA fragments). The length of the tail depends on the number of broken DNA strands [59].

The cells (1 × 10^3^) were seeded in 35 mm dishes and grown in medium for 24 h to allow them to reach the log phase. After 24 h of cell culture, the tested compounds (IC_50_ concentration) were added and cells were incubated with compounds for another 24 h. After this time, the cells were trypsinized, centrifuged, resuspended in a little PBS, and added to low-melting-point agarose solution. A small amount of the agarose suspension of cells prepared in this way was placed on a glass slide, coated with normal melting point agarose, covered with a coverslip, and left until the agarose solidified. The slides were incubated for at least one hour in lysis buffer (2.5 M NaCl, 100 mM, Na_2_EDTA, 10 mM, Tris, 1% Triton X-100) to release DNA. The slides were then washed three times with development buffer (1 mM, Na_2_EDTA, 300 mM NaOH) and incubated in the same buffer for 20 min. The next step was the electrophoresis of cells in electrophoretic buffer for 20 min (29 V, 30 mA). After electrophoresis, the slides were allowed to dry and then stained with DAPI solution (2 mg/mL). The comet DNA analysis was performed under a Nikon Eclipse fluorescence microscope (Nikon, Tokyo, Japan) equipped with a 4910 COHU video camera (Cohu, Inc., San Diego, CA, USA) and a computer with Lucia-Comet v. 4.51 software installed (Imaging Laboratory, Prague, Czech Republic). Each time, from each preparation, 100 randomly selected comets were counted. One obtains an image in the form of “comets”. The “head” is where the cell immobilizes before lysis, the “tail” is identified as damaged DNA fragments. The measure of the level of DNA damage is the length of the tail and the amount of DNA it contains. The slides are analyzed under a fluorescence microscope equipped with a filter adapted to the previously used fluorescent dye. The DNA damage percentage is calculated by Lucia Comet, based on measurements of the color intensity and the length of the comet tail. The intensity of fluorescence is determined by software, which calculates a series of parameters characterizing the amount of DNA damage for each cell. The percentage of DNA in the comet’s “head” corresponds to genetic material located in the nucleus, and the fluorescent region of the “tail” corresponds to the genetic material that has left the cell nucleus during electrophoresis.

### 4.7. Measurement of Cleaved PARP Levels

Cells (1 × 10^6^) were seeded in 35 mm dishes and cultured for 24 h in the appropriate medium until they reached log phase growth. After this time, the analyzed compounds were added to the cells at the IC_50_ concentration and incubated for 24 h. After incubation, the medium was removed, the cells were washed with PBS solution and trypsinized. The harvested cell pellet was lysed in RIPA buffer (50 mM Tris-HCL, pH 8.0, with 150 mM sodium chloride, 1.0% Igepal CA-630 (NP-40), 0.5% sodium deoxychlorate, and 0.1% sodium dodecyl sulfate) with protease inhibitor (phenylmethylsulfonyl fluoride) (Sigma-Aldrich Sigma, St. Louis, MO, USA). The level of cleaved PARP was estimated using the PARP Cleaved [214/215] ELISA kit (Invitrogen, Waltham, MA, USA) according to the protocol described in the manufacturer’s instructions. 

### 4.8. Determination of Apoptotic and Necrotic Cell Fractions by Fluorescence Microscopy (Double Staining of Cells with Fluorescent Dyes Hoechst 33258 and Propidium Iodide)

Live, early apoptotic, late apoptotic, and necrotic cells were identified by the simultaneous use of two fluorescent dyes (propidium iodide and Hoechst 33258; Sigma, St. Louis, MO, USA). Propidium iodide (PI) only penetrates cells with damaged cell membranes, identifying necrotic cells or cells in the late stages of apoptosis, while Hoechst 33258 penetrates freely through the intact membrane of living and early apoptotic cells. As a result of the dye penetrating through intact biological membranes, it stains the DNA of the cell nucleus a light blue color. The dye fluorescence intensity is related to the degree of DNA packing, distinguishing strongly fluorescent apoptotic cells containing highly condensed chromatin from weakly fluorescing living cells containing looser chromatin. Both fluorochromes are excited by ultraviolet light: propidium iodide exhibits orange-red fluorescence, while Hoechst 33258 emits blue light. The combination of dyes allows the following to be identified:

- Live cells (weak, dull light blue fluorescence);
- Cells in the early phase of PCD (bright, light blue fluorescence);
- Cells in the late phase of PCD (pink and purple fluorescence);
- Necrotic cells (intense red fluorescence).

The cells (1 × 10^3^) were seeded in six-well plates and cultured in appropriate culture medium for 24 h until the log phase growth was reached. After this time, the test compounds were added to the culture medium at a concentration of IC_50_. Cells were incubated with compounds for 24 h. After treatment, the medium was removed, the cells were washed with HBSS solution, and then 3 mL of HBSS containing Hoechst 33258 (0.13 mM) and PI (0.23 mM) was added. The cells were incubated in darkness with fluorochromes for approximately five minutes at room temperature. After this time, the HBSS solution containing the fluorescent dyes was removed and fresh HBSS was added to the cells. The microscopic image analysis on the basis of the dye fluorescence was performed using a fluorescence microscope (Olympus IX70, Tokyo, Japan) under 400 magnifications. The cells were classified as live, apoptotic, or necrotic on the basis of their morphological and staining characteristics.

### 4.9. Statistical Analysis 

All data are expressed as mean and SD values, and as percentages of control values (untreated cells; 100%). The normality of data was tested with the Shapiro–Wilk test, and the homogeneity of variance was verified with Levene’s test. The significance of the differences between pairs of means was estimated using one-way ANOVA and the post hoc Tukey’s test. All statistics were calculated with the STATISTICA software package (StatSoft, Tulsa, OK, USA). A * *p* value of <0.05 was considered significant.

### 4.10. Molecular Docking

Although molecular docking studies have been performed on compounds N and S using the Gold program [60] and using the ChemPLP scoring function, iron was used instead of ruthenium. The use of iron complexes as a proxy to ruthenium complexes has been extensively validated elsewhere [61] (in the main text and accompanying SI file); docking an iron complex to an enzyme was found to correspond to previous data regarding a ruthenium complex docked with the aid of the Hex program. Iron is a better candidate, as its parameters have been included in parametrization sets in most docking programs. 

The density functional theory (DFT) calculations were carried out using the Gaussian16 program [62] with ωB97X-D functional [63,64] expressed in the def2-TZVP basis set [65]. The crystal structures of tyrosine hydroxylase (PDB ID: 1TOH [66]), UDP-*D*-glucose-6-dehydrogenase (PDB ID: 4EDF [67]), and the DNA fragment (PDB ID: 1FYY [68]) were long enough to allow for studies of intercalation and binding to the minor groove (all data were retrieved from the protein data bank (http://www.rcsb.org/) (accessed on 3 February 2023) [69]. Since blind docking was not possible in the case of 1TOH, the study examined the dependence of results on the radius (from catalytic iron) of the docking sphere; it was found that the scoring functions are not dependent on the radius. Therefore, both enzymes used a default radius of 10 Å, which proved to be adequate for blind docking in the case of 4EDF (chain A). In the case of 1FYY, the docking conditions comprised spheres with a 7.5 Å radius, centered either at the center of the natively-intercalated ligand, or the spheres with 7 Å radius moved toward the groove, as described previously [61]. The metallo-protease template was used for 1TOH, and the default template for the other two enzymes. Visualization was performed using Chimera [70], Mercury [71], and GaussView Version 6.1; Shawnee Mission, KS, USA [72] software.

### 4.11. Lipophilicity of Chromatography Methods of RP-TLC

All the experimental solutions of standard substances (0.1 mg/mL) were prepared by dissolving ligands (**1a**–**1d**) or ruthenium complexes (**2a**/**3a**–**2d**/**3d**) in ethanol. Commercially-available octadecyl-modified silica aluminum sheets (20 × 20 cm) were cut into 5 × 10 cm plates. The plates were manually spotted with 1.0 μL of freshly prepared solutions using automatic pipets, 5 mm from each edge. The chromatograms for all compounds (**1a**/**2a**/**3a**–**1d**/**2d**/**3d**) were developed using a horizontal developing chamber and the solvent migration distance was about 4.5 cm. The plates were visually inspected under UV light at 254 nm. Each zone was clearly marked and its distance was manually measured. The RP-TLC analyses of lipophilicity were performed at ambient temperature (22 °C). In this experiment, all measurements were performed in triplicate and the obtained results were used in further calculations. The acetonitrile and water mixtures for ligands **1a**–**1d** and arene–ruthenium(II) complexes **2a**/**3a**–**2d**/**3d** were used as mobile phases, with compound content increasing in the range of 30–70% *v*/*v*, at 5% intervals. Values of the chromatographic retention coefficient R_f_ (as shown Formula (1)) and R_M_ (as shown Formula (2)), based on received chromatograms, were calculated and converted as follows:

(1) $$R_{f}=\frac{a}{b}$$

(2) $$R_{M}=\log\left(\frac{1}{R_{f}}-1\right)$$

### 4.12. The pH Dependent of Stability of the Complexes

The stability measurements were performed on a UV 1800 Shimadzu spectrophotometer (Kyoto, Japan) at room temperature. The complexes **2d** and **3d** were tested for the stabilities in buffer Na_2_HPO_4_ (pH 4 and 8). UV–Vis spectra were recorded hourly for 48 h at 22 °C. The spectra were recorded in the range of 200–800 nm over a time course of 0, 24, and 48 h.

## 5. Conclusions

Eight *p*-cymene–ruthenium(II) complexes were tested for their potential anticancer properties against fifteen human cancer cell lines. The selected compounds showed strong anti-cancer properties with a high selectivity of biological action against normal endothelial cells.

The arene–ruthenium(II) complexes (**2a**/**3a**–**2d**/**3d**) induced the apoptotic pathway in cancer cells. One of their mechanisms of cytotoxic activity was the generation of ROS/RNS, which, as highly reactive molecules, destroyed cellular organelles (e.g., peroxidation of membrane lipids) and led to the death of cancer cells. Moreover, all ruthenium(II) complexes were characterized by high genotoxicity, resulting in significant damage to the DNA of cancer cells after exposure. The repair of DNA damage was, in turn, effectively inhibited by the ruthenium-mediated degradation of PARP-1 polymerase. The results obtained in vitro and during in silico molecular docking indicate a high affinity of the analyzed derivatives for PARP1 polymerase and its degradation.

Based on the conducted studies, we certainly cannot indicate clear differences in the biological anticancer activity of compounds containing [Cl]^−^ ions (**2a**–**2d**) and compounds containing [PF_6_]^−^ ions (**3a**–**3d**), especially in such extensive studies using many different types of cancer cells. These complexes can demonstrate increased solubility and greater bioaccumulation in biological systems by exchanging ions. However, the obtained data allow us to conclude that in addition to the relationship between the structure of the compound and its biological activity, the type of cancer cell and its origin are also of key importance regarding arene–ruthenium(II) complexes with carbothioamidopyrazoles as ligands.

In our future research, we would like to focus on a detailed analysis of the molecular activity of the tested ruthenium derivatives, taking into account a specific type of cancer. The combination of arene–ruthenium(II) complexes with carbothioamidopyrazoles ligands may be a promising strategy for the creation of a potential multifunctional heteronuclear metal ion complex for use in anticancer therapies.

## Figures and Tables

**Table 1:** In vitro cytotoxicity of newly synthesized compounds **1a/2a/3a**–**1d**/**2d**/**3d** and dimer dichloro(*p*-cymene)ruthenium(II) **(4)** against various human cancer cells. The cells were incubated with the compounds for 48 h *.

Cell Line	Cytotoxic Effect of Investigated Compounds. IC_50_ (µM)												
	1a	1b	1c	1d	2a	2b	2c	2d	3a	3b	3c	3d	4
HT29	>200	>200	>200	>200	111.3 ± 4.9	106.6 ± 5.9	29.5 ± 2.1	168.1 ± 7.5	123.3 ± 3.9	122.8 ± 3.7	128.1 ± 4.2	185.2 ± 1.8	>200
Colo205	>200	>200	>200	>200	109.8 ± 6.7	114.6 ± 4.1	32.6 ± 3.3	>200	59.6 ± 3.4	91.6 ± 1.6	26.7 ± 2.1	>200	>200
SW620	>200	>200	>200	>200	117.6 ± 6.1	107.3 ± 3.4	49.4 ± 1.2	>200	63.9 ± 2.7	78.8 ± 7.4	90.1 ± 1.7	>200	>200
LoVo	>200	>200	>200	>200	>200	59.7 ± 3.2	42.6 ± 3.2	>200	126.7 ± 3.7	99.1 ± 9.4	32.4 ± 1.8	110.3 ± 6.9	167.6 ± 7
Caco2	>200	>200	>200	>200	>200	>200	>200	>200	107.2 ± 8.4	82.4 ± 8.4	172.2 ± 8.2	161.9 ± 6.1	>200
Hep 3b	>200	>200	>200	>200	>200	>200	104.2 ± 4.4	>200	55.3 ± 2.6	62.4 ± 2.7	52.1 ± 3.4	>200	166.6 ± 6.8
HEC1A	110.8 ± 9.6	114.4 ± 11.3	109.5 ± 8.9	99.1 ± 5.7	62.3 ± 0.9	36.4 ± 1.7	24.5 ± 0.9	67.8 ± 6.1	77.2 ± 4.4	40.1 ± 2.1	39.9 ± 4.1	127.6 ± 2.1	107.8 ± 3.4
Ishkiawa	124.3 ± 4.8	100.1 ± 4.6	94 ± 2.4	>200	90.7 ± 3.7	51.4 ± 1.9	142.3 ± 7.4	100 ± 6.7	>200	121.4 ± 3.7	124.3 ± 0.9	>200	120.1 ± 7.2
Hela	>200	>200	>200	>200	>200	164.5 ± 8.5	124.3 ± 7.2	111.3 ± 1.7	98.7 ± 7.4	101.2 ± 6.7	78.8 ± 1.1	>200	>200
A549	>200	>200	>200	>200	120.7 ± 3.6	>200	>200	>200	>200	162.4 ± 7.9	191.1 ± 4.3	152.2 ± 2.8	>200
HCC38	>200	>200	>200	>200	84.4 ± 1.1	63.7 ± 4.4	>200	>200	>200	>200	131.4 ± 2.2	187.6 ± 6.4	>200
MCF7	>200	>200	>200	>200	>200	82.7 ± 3	83.4 ± 7.3	>200	>200	132.4 ± 5.3	30.4 ± 2.1	>200	>200
WM115	>200	>200	>200	90.9 ± 10.2	60.2 ± 6.3	54.9 ± 5.9	26.7 ± 3.3	8 ± 0.9	64.1 ± 5.1	51.7 ± 5.3	18.68 ± 1.7	>200	>200
NALM6	>200	>200	152.3 ± 26.7	>200	51.6 ± 5.7	40 ± 5.6	11.7 ± 1.6	>200	46.1 ± 5.1	18.5 ± 2.3	19.8 ± 1.8	>200	>200
HL60	>200	>200	>200	>200	88.9 ± 6.1	80.8 ± 3.9	86.5 ± 8	>200	79.7 ± 7.2	52.9 ± 2.1	70.4 ± 6	>200	>200

* Results are expressed as means ± SEM of three repeated experiments.

**Table 2:** In vitro cytotoxicity of reference cisplatin and NAMI-A compounds against various human cancer cells. The cells were incubated with the compounds for 48 h *.

Compounds	Cytotoxic Effect of Reference Compounds, IC_50_ (µM)									
	NALM6	HEC1A	WM115	COLO205	Hep 3b	HT29	HCT116	SW620	Ishikawa	MCF7
Cisplatin	0.7 ± 0.3	89.4 ± 4.7	16.9 ± 4.3	26.7 ± 4.2	24.3 ± 2.9	141.3 ± 7.4	37.1 ± 2.8	26.4 ± 6.3	16.1 ± 2.5	11.4 ± 1.3
NAMI-A	>200	>200	>200	>200	>200	>200	>200	>200	>200	14.1 ± 1.5

* Results are expressed as means ± SEM of three repeated experiments.

**Table 3:** In vitro cytotoxicity of newly synthesized compounds **2a**–**3c** against normal, immortalized human microvascular endothelial cell line HMEC1. The cells were incubated with the compounds for 48 h *.

Cell Line	Cytotoxic Effect of Investigated Compounds, IC_50_ (µM)						
	2a	2b	2c	2d	3a	3b	3c
HMEC1	156.1 ± 4.9	124.6 ± 2.1	191.7 ± 6.5	163.0 ± 5.9	>200	134.8 ± 6.7	164.5 ± 3.1

* Results are expressed as means ± SEM of three repeated experiments.

**Table 4:** Docking results.

Compounds	Conformers	1TOH	4EDF	1FYY Intercallation	1FYY Minor Groove
2a	N17	50.4	47.0	49.9	35.2
	S17	57.8	62.8	55.2	46.0
2b	N18	57.0	50.7	52.1	33.8
	S18	69.0	73.6	59.5	45.2
2c	N1	57.2	60.3	61.5	42.3
	S1	73.2	77.9	66.7	46.8
2d	N2	52.3	46.4	42.6	37.9
	S2	62.3	64.1	52.1	47.1

**Table 5:** Lipophilicity values for ligands **1a**–**1d** and their complexes **2a/3a**–**2d/3d**.

Compounds	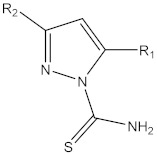 **L**	R_M_
**1a**	L1: R_1_ = –CH_3_; R_2_ = –CH_3_	2.32
**1b**	L2: R_1_ = –CH_2_CH_3_; R_2_ = –CH_2_CH_3_	2.51
**1c**	L3: R_1_ = –OH; R_2_ = —C_6_H_5_	2.04
**1d**	L4: R_1_ = –OH; R_2_ = –CH_3_	1.29
**2a**	*p*-cymRuL1Cl	2.40
**2b**	*p*-cymRuL2Cl	2.59
**2c**	*p*-cymRuL3Cl	2.27
**2d**	*p*-cymRuL4Cl	1.64
**3a**	*p*-cymRuL1PF_6_	2.55
**3b**	*p*-cymRuL2PF_6_	2.73
**3c**	*p*-cymRuL3PF_6_	2.61
**3d**	*p*-cymRuL4PF_6_	2.39

Eluent AcCN: H_2_O at 30–70% *v*/*v* concentration was used for ligands **1a**–**1d** and complexes **2a**/**3a**–**2d**/**3d**.

## Data Availability

Data are contained within the article.

## Sample Availability

Samples of the compounds **1a**/**2a**/**3a**-**1d**/**2d**/**3d** are available from the autor E. Namiecińska.
